# Computational methodology for the development of microdevices and microreactors with ANSYS CFX

**DOI:** 10.1016/j.mex.2019.12.006

**Published:** 2019-12-10

**Authors:** Harrson S. Santana, Adriano G.P. da Silva, Mariana G.M. Lopes, Alan C. Rodrigues, Osvaldir P. Taranto, João Lameu Silva

**Affiliations:** a School of Chemical Engineering, University of Campinas, Zip Code 13083-852, Campinas, SP, Brazil; b Federal Institute of Education, Science and Technology of South of Minas Gerais, Zip Code 37560-260, Pouso Alegre, MG, Brazil

**Keywords:** Santana/Silva Jr. computational methodology, Microfluidics, Microreactor, Numerical simulation, Process optimization, Computational fluid dynamics

## Abstract

In the present paper, a computational methodology for the development of microdevices and microreactors are presented. The methodology was based on Computational Fluid Dynamics (CFD), i.e., the solution of transport equations governing the flow field. The methodology presents a step-by-step tutorial for modeling and simulation of such microdevices that can be used by beginner or experienced users. The proposed methodology was employed in two study cases: fluid mixing and fluid mixing accompanied by chemical reaction. Two new geometry designs were evaluated: a micrometric scale channel with triangular cross section (MT) and a millimeter range scaled channel (MTB). It is expect that the reported methodology contributes to the popularization of CFD usage among researchers, scientists and Microfluidic enthusiasts. Also, it can motivate future studies to focus firstly on geometry optimization by numerical simulations, providing a faster and economical way to develop microdevices.

• A CFD-based methodology was presented for the development of microdevices and microreactors
• The methodology can be used in distinct fluid mixing and chemical reaction systems
• Numerical simulation allows a faster microdevice development procedure with costs reductions

## Graphical abstract

**Figure:**
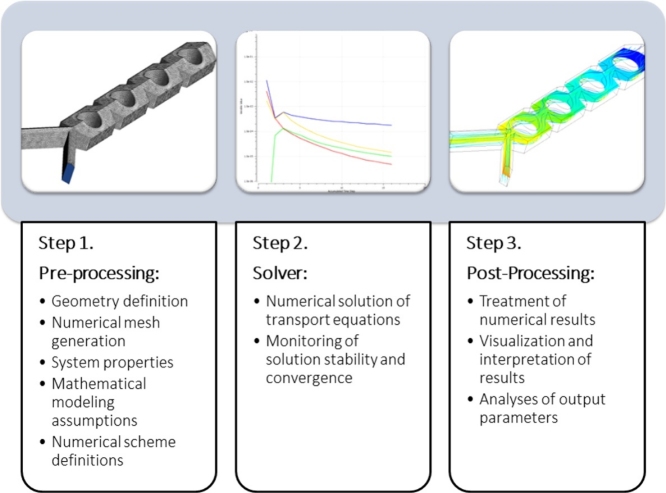

**Specification Table**

**Table:** 

Subject Area:	Engineering
More specific subject area:	Development of microdevices and microrreactors using Computational Fluid Dynamics
Method name:	Santana/Silva Jr. computational methodology
Name and reference of original method:	
Resource availability:	For the methodology reproduction the software ANSYS ® Meshing and ANSYS ® CFX, version 19 was employed, available at: https://www.ansys.com/products/fluids/ansys-cfx https://www.ansys.com/products/platform/ansys-meshing

## Method details

### Introduction

Computational Fluid Dynamics (CFD) is a numerical technique used in analyses of flow system and associated phenomena. CFD can be understood as a combination of physics, calculus and computational sciences, aiming the flow field description [1,2]. A CFD solver is based on the solution of continuity and momentum balance equation. Other physical and physico-chemical phenomena can be coupled in the simulations, for example, heat and mass transfer and chemical reactions (homogeneous or heterogeneous systems). CFD became an essential tool for engineers mostly in design and optimization of process and equipment, once it presents some advantages such as costs and time reduction of projects (e.g., simulation and analysis of several design before manufacturing of physical prototypes), great level of flow field details and prediction of flow field in severe operation conditions, where physical measurement are very expensive or even impossible. On the other hand, Verification and Validation (V&V) steps are essential for the mathematical model reliability (CFD simulations could reach numerical convergence, however, only this mathematical convergence do not ensure the realistic physics). The verification step consist in evaluate the real behavior of the studied phenomena, while, the validation step consists in the comparison between numerical predictions with experimental data or analytical solutions, when possible [3,4]. Accordingly, CFD is a powerful tool used in several areas of human knowledge, including chemical, mechanical, aerospace and biomedical and recently in Microfluidics.

Microfluidics can be defined as the science and technology handling flows in micrometric structures. The main advantages of microscale are the easiness of temperature control, minimization of subproducts, low amount of reactants and samples, short residence times to achieve the required reactor performance, high surface area-to-volume ratio, high heat and mass transfer rates and improved safety conditions in processing of flammable, toxic and explosive substances. In order to design and optimize microdevices, CFD appears and interesting tool. Hessel et al. [5] reported that the modeling and simulation of microdevices was still a new subject regarding conventional scale. Although modeling and simulation has recently become more common among researches in Microfluidics, the microdevice project is mostly defined from literature review and experimental background.

Therefore, in the present paper was reported a computational methodology for the development of microdevices and microreactors that could be extended to other microfluidic devices considering its specific characteristics. The reported methodology presents a step-by-step methodology for modeling and simulation of microdevices that could be used by beginners or experienced professionals. Furthermore, the methodology was applied in two study cases: fluid mixing (Alcohol/Sunflower oil) and fluid mixing accompanied by chemical reaction (Biodiesel synthesis). Despite the methodology was developed based on biodiesel synthesis, the reader could adapt it for other chemical reaction and fluid mixing systems following the described considerations.

### Computational Fluid Dynamics in the development of optimal microdevices

**Fig. 1:**
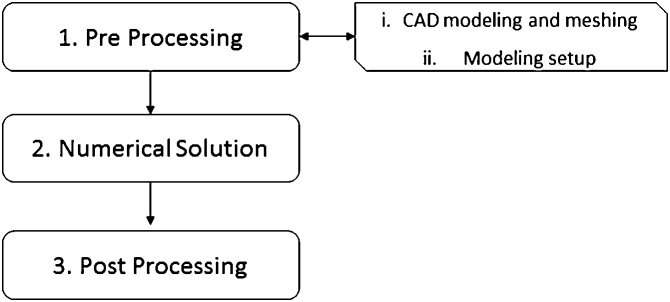
Flowchart of Computational Fluid Dynamic analysis.

CFD simulations can be divided in three main steps, as depicted in Fig. 1: 1 - pre-processing (definition of the region of interest, numerical mesh generation, initial and boundary conditions definitions, mathematical modeling and numerical schemes definitions); 2 - simulation solving (numerical solution of the transport equations system); 3 – post-processing (treatment and analysis of results).

Wilcox [6] pointed out three essential keys to perform reliable CFD simulations: 1 – numerical mesh generation; 2 – turbulence modeling; 3 – algorithms of solution. The mesh generation integrates the first step of a CFD analysis. This procedure consists in the subdivision of the geometry in a finite number of control volumes (CV). To ensure the quality of the mesh spatial resolution two methods are usually employed. The first one is known as “numerical independence mesh test”. This method consists in simulate a case of study using several meshes (usually about 4–6 meshes are sufficient) with different spatial resolution (increasing the refinement). The results are compared, for example, a velocity profile or a pressure drop, evaluating the converge among these meshes. The numerical mesh selection is based on the spatial resolution where significant differences in the results vanish or tend to be negligible. Thus, the smaller resolution which provides an analogue detailing of the most refined mesh are used. Another methodology is the Grid Convergence Index (GCI) described by Celik et al. [7], a well-accepted method for numerical simulation studies. In general, this method is based on the simulation of three meshes with different spatial refinement. The results of these meshes are compared and extrapolated values are obtained from Richardson extrapolation procedure. Based on this, the numerical uncertainties from spatial discretization are estimated. The time dependence of the numerical solutions (for transient cases) is based on the non-dimensional Courant number, defined as the ratio of time step to the characteristic convective time [8].

The second point highlighted by Wilcox [6] is the turbulence modeling. For turbulent regime flows three approaches (Reynolds Averaged Navier-Stokes – RANS, Large Eddy Simulation – LES, Direct Numerical Simulations, DNS), can be employed in accordance to the required detail level of the fluid dynamics and the available computational power resources. Especially for microscale application, it is important to point out that the prevailing flow regime is laminar due to the small structure dimensions. For this flow regime, the exact form of Navier-Stokes equations is numerically solved in space and time (transient cases) and space (steady-state cases). This particularity provides superior reliability for the flow field prediction, since the additional detailed investigation and validation of the turbulence modeling are not necessary for these cases.

The last essential key is the algorithm of solution. The most part of the available computational codes use the Finite Volume Method (FVM). This method uses the integral form of the conservation equations as a starting point. These equations are solved at the CV nodes (central point of each CV) and demand interpolation schemes in order to estimate the transported quantities at CV faces (required to calculate the fluxes through the control surfaces of CV). Mathematical formulations for the approximation of face to nodal value have received great attention and are well-established and validated [8]. As result, the FVM results in an algebraic equation system, which can be solved by algorithms, as for example, the SIMPLE or PISO [4] implemented in the most of available CFD codes.

**Fig. 2:**
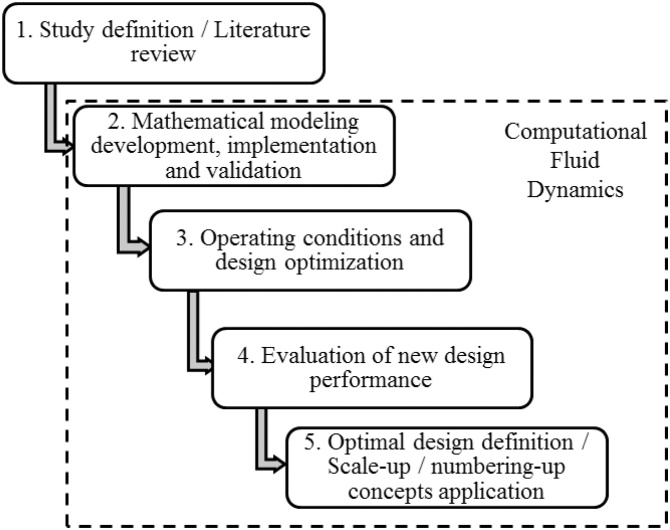
Proposed methodology for process optimization using CFD.

In order to employ the CFD tool for development and optimization of microdevices, a methodological approach is presented in Fig. 2 and detailed as follows:

• Step 1 - Problem Statement: Definition of the process to be optimized, carrying out a state-of-art literature review, pointing out the main spots (advantages, disadvantages, challenges to overcome) to be approached;
• Step 2 - Mathematical model development, implementation and validation: The main physical and physicochemical characteristics must be considered, as for example, mass transfer, chemical kinetics coupling in fluid dynamic model. The validation with experimental data and/or analytical solution (when possible) is fundamental to ensure the prediction reliability of the proposed model.
• Step 3: Based on the most influential operating variables, the operating conditions could be optimized to improve the process performance. This step can be performed jointly with a Design of Experiments. After the mathematical model validation (Step 2) and the operating conditions optimization (Step 3), new geometry designs can be proposed and promptly developed using CAD modeling, following by mesh generation and simulation.
• Step 4: The new design performance evaluation must be carried out using representative output parameters. In order to study the micromixer optimization, the mixing degree, a quantitative parameter of the mixing quality between the reactants species, ranging from zero (total segregation of species) and one (complete/ideal mixing of species) – detailed in Santana et al. [9], is an interesting output parameter. Another important parameter is the pressure drop or even a correlation between pressure drop and mixing degree or reactive efficiency (for chemical reactive processes).
• Step 5: After the numerical analysis of operating conditions optimization and new design development, the definition of the optimized setup could be performed. The scale-up or numbering-up concepts could be carried out using the optimal design, to increase the process throughput. The numbering-up concept represent a smart alternative to reach higher capacities using microdevices. In this type of process scaling, the optimized microunit is multiplied to operate continuously in parallel. The main advantage is the flexible operation regarding production and maintenance. In the case of a unit exhibit failure, it can be fixed without stopping the entire microplant, keeping other microunits in continuous operation [[10], [11], [12]]. In order to achieve higher throughput, several modules of the numbered-up parallel microdevices can be used. This new type of continuous and modular operation offers opportunities for process intensification with different investments risks [13].

### Mathematical modeling and numerical details

The fluid dynamic and chemical reaction studies were accomplished using the computational code ANSYS CFX 19. It is based on the solution of conservation equations of the transported properties, using an Element-Based Finite Volume Method in the numerical procedure. This CFD package was preferred due to the satisfactory performance on modeling multicomponent flows in microscale as seen in previous studies [[14], [15], [16],^9^]. In addition to features such as user-friendly interface, easiness on implementing new materials and source terms, good convergence and numerical stability of such simulations were also taken into account. In this research, the conservation of total mass (continuity) (Eq. (1)), momentum (Navier-Stokes) (Eq. (2)) and mass of chemical species (Eq. (3)) were solved for incompressible, steady-state, isothermal and laminar flow conditions:

(1) ∇⋅U=0

(2) $$\rho \;\left(U\cdot \nabla U\right)=-\nabla p+\mu \nabla^{2}U+\rho \;g$$

(3) $$\rho \left(\;U\cdot \nabla Y_{i}\right)=\rho \;D_{i}\;\nabla^{2}Y_{i}+S_{i}$$

where *ρ* is the specific mass (kg m^−3^), *U* is the velocity vector (m s^-1^), μ is the dynamic viscosity (Pa s), *g* is the gravity acceleration (m s^-2^), *p* is the pressure (kg m^-1^ s^-2^), *Y* is the mass fraction, *D_i_* is the kinematic diffusion coefficient (m^2^ s^-1^) and *S* is the mass source due to chemical reactions (kg m^−3^ s^-1^), modeled in accordance with Eq. (4):

(4) $$S_{i}=\left(\sum_{r_{r}}^{n}\nu_{i}^{''}r_{r}-\sum_{r_{r}}^{n}\nu_{i}^{'}r_{r}\right)\;M_{Wi}$$

where *M_W_* is the molecular weight, *ν’’* e *v’* are the stoichiometric coefficient of the chemical species as products or as reactants, respectively, at the chemical reaction *r* and *r_r_* is the rate of reaction *r*.

The mass diffusion coefficient of ethanol (1.2 × 10^−9^ m^2^ s^-1^) was estimated using the Wilke-Chang correlation [25], considering ethanol in sunflower oil. For the other species, the mass diffusivity coefficients were estimated based on their viscosities using ethanol-oil diffusion coefficient as reference [17]. The chemical reactions were considered to be in equilibrium state, i.e., under steady state conditions, denoting that the reaction rate of reagents to products, as well as the reaction rate of products to reagents, in each reaction step was constant over time.

For the numerical solution, high order discretization schemes were employed. A convergence criteria of RMS = 1 × 10^−6^ was defined for a solving iteration range of 500-5000. The simulations were solved in parallel processing using computer nodes with 8 Intel Xeon 3 GHz, 16GB RAM processors using Linux Suse 64-bit OS. The advection terms of transport equation were interpolated by a high order Upwind scheme. Shape functions were employed to interpolate diffusion and pressure gradient terms. These functions were derived from Finite Element Method, being used to estimate the flow properties values inside the control volume. In general, the Ansys CFX uses a tri-linear shape function based on parametric coordinates. The transported property value, *Φ*, of a control volume is then given by the summation over all control volumes nodes of the product between the shape function value and the nodal value (*Φ_CV_ =* Σ*N_i_.Φ_i_*, where *Φ_CV_* is the control volume transport property, *N_i_* is the shape function value - *N_i_* = 1, if *i = j* and *N_i_* = 0 if *i ≠ j -* and *Φ_i_* is the nodal value of transport property). The ANSYS CFX employs a coupled solver for the transport equation system, based on Rhie and Chow [18] discretization method modified by Majumdar [19] in order to eliminate the time-step dependence for steady-state simulations. The Algebraic Multigrid Method was used to improve the convergence in the linear equation system solution procedure. This method provides a virtual coarsening of mesh spacing during the iteration process, then, a re-refinement is performed to achieve an accurate solution [20].

The boundary conditions employed in the numerical solution of transport equations are described below:

• Inlet 1: Alcohol feed stream, with pure alcohol flowing into domain (*Y_A_=* 1*; Y_TG_=* 0 or *Y_W_=* 0), with uniform velocity normal to boundary inlet surface (*u = U_A_*);
• Inlet 2: Sunflower oil feed stream. Pure oil (triglyceride) stream flowing into domain (*Y_TG_=* 1*; Y_A_=* 0), with uniform velocity normal to boundary inlet surface (*u = U*).

The inlet velocities were defined based on Reynolds number (Eq. (5)) and ethanol/oil molar ratio. For mixing studies, the fluid velocities were considered equal with Reynolds number obtained from sunflower oil. For the chemical reaction analysis, the ethanol/oil molar ratio was considered to determine the inlet velocities.

• Outlet: Outlet condition with zero relative pressure;
• Walls: Solid stationary surface with no-slip condition.

(5) Re=u dHν

where *d_H_* is the hydraulic diameter (m) and *ν* is the kinematic viscosity (m^2^ s^−1^), the subscripts *TG* and *A* indicate triglycerides and alcohol.

### Reaction kinetics

Transesterification reaction can be described by a series of three consecutive reversible reaction steps according to Eqs. (6), (7), (8). In general, the tri- (TG), di- (DG) and mono- (MG) glycerides combine with an alcohol molecule (A) in alkaline medium at each step, producing an ethyl ester (E) molecule, until it finally produces glycerol (*GL*) [21]:

(6) TG+A↔DG+E

(7) DG+A↔MG+E

(8) MG+A↔GL+E

The overall biodiesel synthesis reaction expression is given by Eq. (9):

(9) TG+3A↔GL+3E

Marjanovic et al. [26] proposed an expression for the triglyceride reaction rate at equilibrium state, according to Eq. (10):

(10) $$-r_{TG}=-\frac{dC_{TG}}{dt}=\vec{k}C_{TG}C_{A}-\overset{⟵}{k}C_{GL}C_{E}$$

where *–r* is the reaction rate (mol m^−3^ s^-1^), $\vec{k}$ and $\overset{⟵}{k}$ are the reaction rate constants for direct and reverse reactions (m^3^ mol^-1^ s^-1^) respectively, *C* is the molar concentration (mol m^−3^), and the subscripts *TG, A, GL* and *E* denote triglyceride, alcohol, glycerol and ethyl ester species. The kinetic data employed in simulations are given in Table 1.

**Table 1:** Kinetic data employed in the numerical simulations.

Temperature (°C)	Ethanol/oil molar ratio	NaOH concentration (wt. %)	$\vec{k}$ x 10^6^ (m^3^ mol^−1^ s^−1^)	$\overset{⟵}{k}$ x 10^8^ (m^3^ mol^−1^ s^−1^)
50	9:1	1.00	15.68	9.42

The mass conservation of chemical species, based on the overall chemical reaction (Eq. (9)), are described by Eqs. (11), (12), (13), (14):

(11) $$\rho \left(\;U\cdot \nabla Y_{TG}\right)=\rho \;D_{TG}\;\nabla^{2}Y_{TG}+\left(r_{TG}\right)M_{WTG}$$

(12) $$\rho \left(\;U\cdot \nabla Y_{A}\right)=\rho \;D_{A}\;\nabla^{2}Y_{A}+\left(3r_{TG}\right)M_{WA}$$

(13) $$\rho \left(\;U\cdot \nabla Y_{GL}\right)=\rho \;D_{GL}\;\nabla^{2}Y_{GL}+\left(-r_{TG}\right)M_{WGL}$$

(14) $$\rho \left(\;U\cdot \nabla Y_{E}\right)=\rho \;D_{E}\;\nabla^{2}Y_{E}+\left(-3r_{TG}\right)M_{WE}$$

For the reactive system, inert oil (with similar physical properties of sunflower oil) was added in system as a constraint, in order to ensure the mass fraction restriction (Eq. (15)):

(15) $$\sum_{i}^{n_{i}}Y_{i}=1\;∴\;Y_{INERT\;OIL}=1-Y_{TG}-Y_{A}-Y_{GL}-Y_{E}$$

### Mixing index (M), performance index (PI) and reaction efficiency

The micromixer performance was evaluated using the mixing index (*M*) or mixing quality, [22], the performance index (*PI*) and the vegetable oil conversion [14]. The mixing degree between the fluid 1 (sunflower oil) and the fluid 2 (ethanol) was determined based on the mass fraction standard deviation of the fluid 1 in a cross section normal to the flow direction according to Eq. (16):

(16) $$\sigma =\sqrt{\frac{\sum {\left(Y_{i}-\bar{Y}\right)}^{2}}{N}}$$

where *σ* is the mass fraction variance, *Y_i_* is the mass fraction at sampling point *i*, $\bar{Y}$ is the mass fraction average and *N* is the number of sampling points in the cross section plane (over 1000 for the present study). The fluid mixing efficiency was calculated from Eq. (17):

(17) $$M=1-\sqrt{\frac{\sigma^{2}}{\sigma_{\max}^{2}}}$$

where *M* is the mixing index and σ^2^_max_ is the maximum variance over the data interval (mass fraction variance in a cross section plane at fluid inlet). The mixing index is a unity for complete fluid mixing and zero for a complete segregation of fluids.

The mixing efficiency was also evaluated using the Performance Index (*PI*). An efficient micromixer should provide high mixing index with low pressure drop. The *PI* is defined as the ratio of mixing index to the unit pressure drop required according to Eq. (18):

(18) $$PI=\frac{M}{\Delta P}$$

where Δ*P* is the pressure drop along the mixing channel, in Pa.

The oil conversion or the fraction due to the chemical reaction was determined from Eq. (19):

(19) $$\text{Oil Conversion}(\%)=\left(\frac{C_{TG,0}-C_{TG,f}}{C_{TG,f}}\right)$$

where *C_TG,0_* is the initial oil concentration (at Inlet 2) and *C_TG,f_* is the final oil concentration (at Outlet).

The mixing and performance indexes analyses were carried out for a Reynolds number range of 0.01–200 in both designs (micrometric scale channel with triangular cross section (MT) and a millimeter range scaled channel (MTB). The biodiesel synthesis performance was evaluated ranging the residence time of the fluids from 5 s to 120 s.

## Methodology for simulation of microdevices and microreactors

### Geometry and numerical mesh

**Fig. 3:**
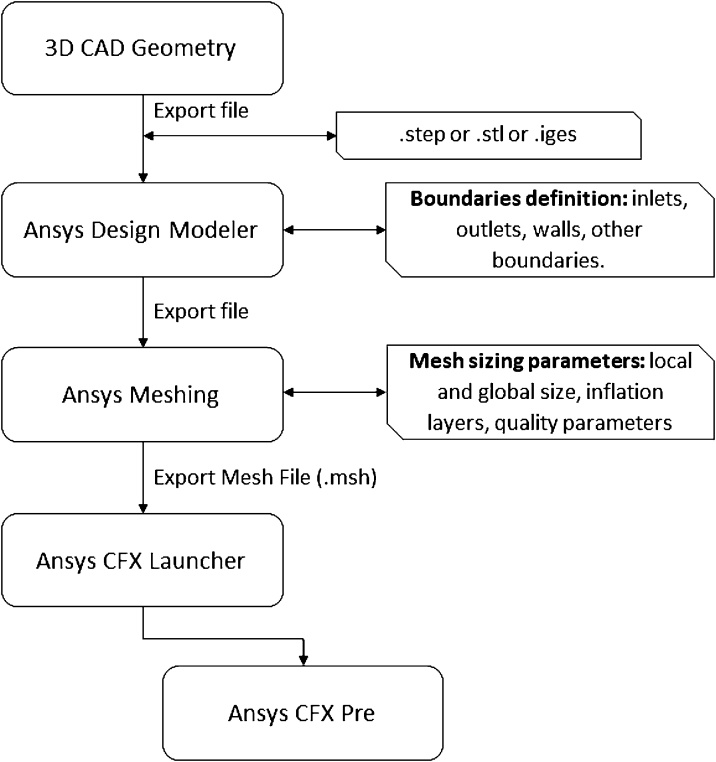
Flowchart of the first part of pre-processing: CAD modeling and mesh generation.

Fig. 3 presents the flowchart summarizing the main steps of pre-processing step as described in the following sections.

#### Importing the external CAD geometry

• Open Ansys Workbench.
• Create a New Project and in the toolbox, create a standalone system selecting the Meshing module from Component System tree. Alternatively, the user can select Fluid Flow (CFX) from Analysis System tree, this option enables to manage all analysis steps from one standalone module.
• Double click on the Geometry to start the Design Modeler.
• In the Design Modeler import the external CAD geometry by clicking: File, Import External Geometry File (in this first example, select the MTB.iges). Alternatively, other CAD output formats can be used, including. STEP and. STL. The main features of these three formats are described as follows: File. IGES: the geometry information is exported as surfaces, wireframe models or solid objects in vector format. IGES extension is a standard format for CAD modeling. The file can be modified downstream the exportation step. File. STEP: the geometry information is exported as standard format to be used in different CAD platforms, avoiding issues related to file exportation errors. The geometry can be modified downstream the exportation, being useful for design procedure. File. STL: this format is most commonly used for 3D printing, presenting high resolution of the surfaces. Depending on the software used downstream of exportation, the geometry cannot be modified.
• Click on Generate, the CAD geometry will be exhibited.
• Save the project.

The CAD model was built in the Autodesk Inventor. The reader can use his preferred CAD software, since the file exportation options support. iges or. step file format, or another format compatible with Ansys Design Modeler. The MTB geometry was based in a previous project of our research groups [14]. The MTB has the dimensions of: 5000 μm (width); 3000 μm (height); 35 mm (longitudinal length). The geometry file (.iges) can be downloaded for free from Supplementary material 1.

#### Defining the boundary conditions before the meshing procedure

**Fig. 4:**
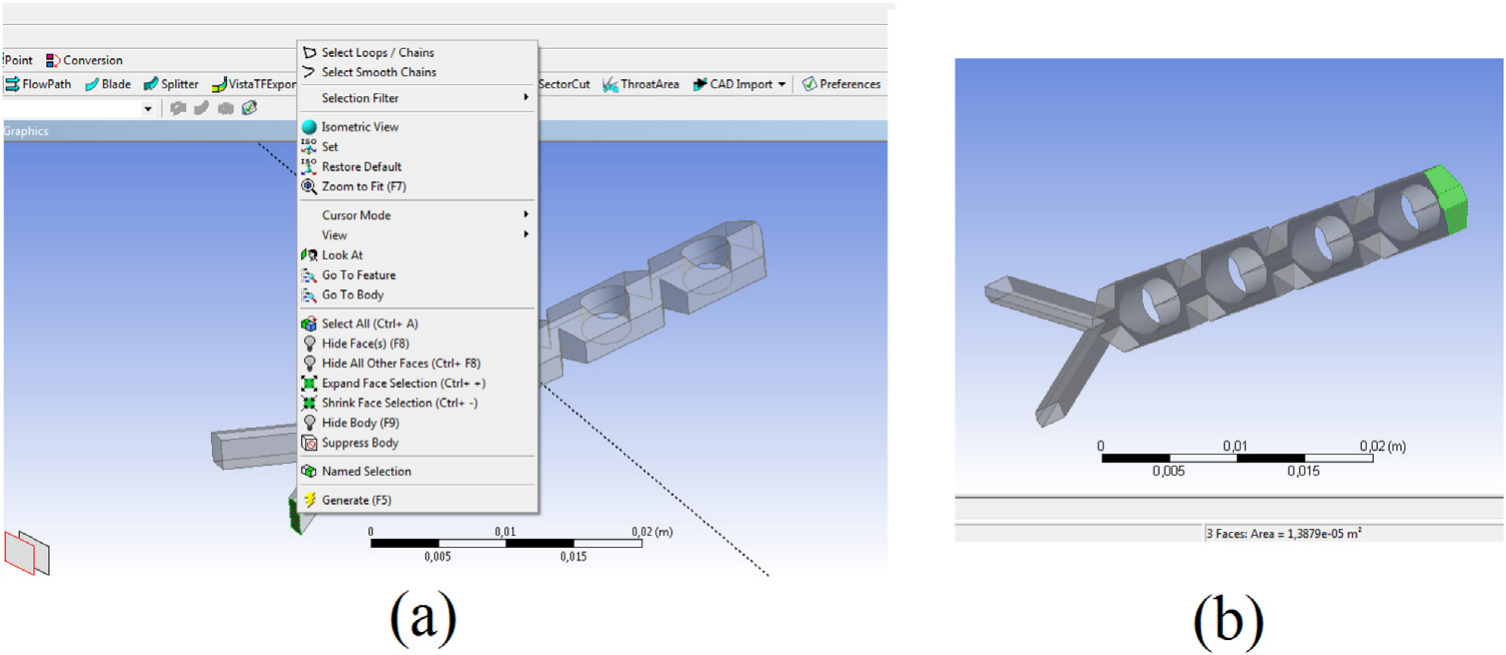
(a) Screen selection of the inlet 1 to create a Named Selection; b) multiple selection of the surfaces corresponding to the outlet boundary condition.
  ○ Rename the selection in the Named Selection line to Inlet-1.
  ○ Click on Generate.
• Inlet2 boundary:
  • Right-click on the surface corresponding to the inlet 2 boundary, then select Named Selection and on the Details View tab, Geometry line, click on Apply.
  • Rename the selection in the Named Selection line to Inlet-2.
  • Click on Generate.
• Outlet boundary:
  • Right-click on the surface corresponding to the outlet boundary, then select Named Selection and on the Details View tab, Geometry line, click on Apply.
  • Rename the selection in the Named Selection line to Outlet.
  • Click on Generate.
• Internal wall boundary:
  • Right-click on the all surfaces corresponding to the internal circular obstacles boundary (use the shift key), then select Named Selection and on the Details View tab, Geometry line, click on Apply.
  • Rename the selection in the Named Selection line to Circular-Obstacles.
  • Click on Generate.
• Wall boundary:
  • Right-click on the Inlet-1 region in the left tree and select Hide Face(s).
  • Right-click on the Inlet-2 region in the left tree and select Hide Face(s).
  • Right-click on the Outlet region in the left tree and select Hide Face(s).
  • Right-click on the Circular-Obstacles region in the left tree and select Hide Face(s).
  • Using CTRL + A, select all remaining surfaces corresponding to wall boundary condition.
  • Right-click on the highlighted surfaces corresponding, select Named Selection and on the Details View tab, Geometry line, click on Apply.
  • Rename the selection in the Named Selection line to Walls.
  • Click on Generate.
• Save the project.
• Close the Design Modeler.

• Inlet1 boundary:
  ○ Right-click on the surface corresponding to the inlet 1 boundary (Fig. 4), then select Named Selection and on the Details View tab, Geometry line, click on Apply.

#### Meshing setup

##### Global mesh size setup

• On the Ansys Workbench project schematic sheet, double click on the Mesh module.
• In Details of Mesh tab perform the following definitions:
  ○ In Defaults, Physics Preference line, select CFD.
  ○ In Defaults, Solver Preference select CFX.
  ○ In Defaults, Element Order select Quadratic.
  ○ In Defaults, Element Size define 0,0005 (1/2 size the smallest inlet channel dimension) (the Mesh module works in SI units by default - it can be changed in Units menu).
  ○ In Sizing, Use Adaptive Sizing, select Yes.
  ○ The remaining parameters can be set up to default values.

##### Local mesh size setup: prism layers and local refinement

• Prism layers around the circular obstacles:
  ○ Right-click on Mesh, chose Insert, Inflation.
  ○ In Details of Inflation tab perform the following definitions:
    ○ Select the Geometry, clicking on it and Apply in Scope, Geometry line.
    ○ In the Scope Method select Named Selection, selecting Circular-Obstacles, click on Apply.
    ○ The remaining parameters can be set up to default values.
• Local refinement on inlet and outlet boundaries:
  • Right-click on Mesh, chose Insert, Sizing.
  • In Details of Face Sizing tab perform the following definitions:
  • Select the inlet and outlet surfaces, click on Apply in Scope, Geometry line.
  • In Definition, set the Element size to 0,00025 (1/2 size the global size defined previously).
  • The remaining parameters can be set up to default values.
  • In the Scope Method select Named Selection, selecting Circular-Obstacles, click on Apply
  • In Definition, set the Element size to 0,00025 (1/2 size the global size defined previously).
  • The remaining parameters can be set up to default values.
• Click on Generate Mesh.
• Save the project.

##### Checking the mesh quality

**Fig. 5:**
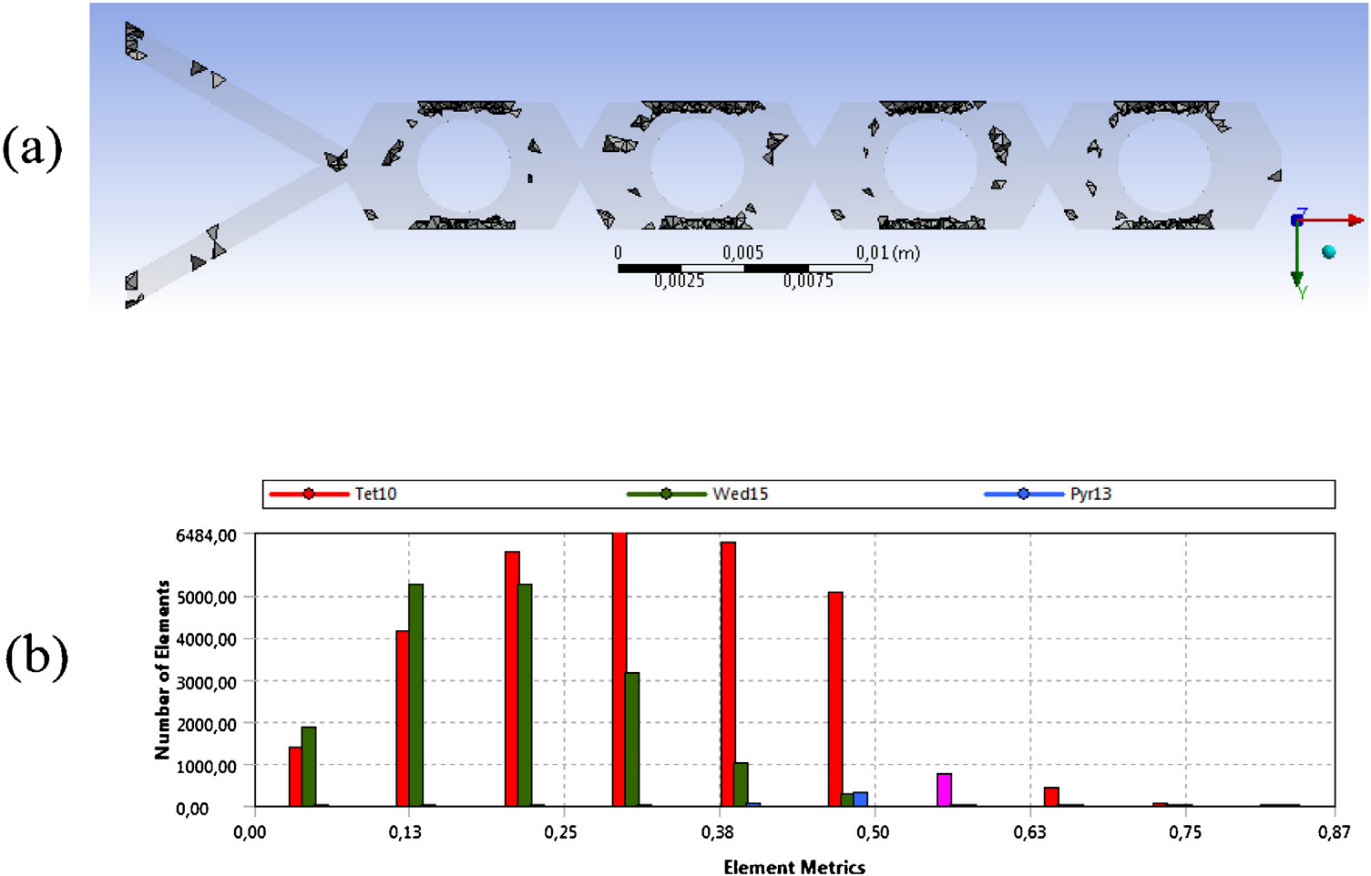
(a) Highlighted elements corresponding the histogram bar selection of skewness about 0,565; (b) histogram showing the distribution of number of elements to the corresponding skewness.
• Export the created mesh (Fig. 6) to Fluent format, clicking on File, Export, in type select FLUENT input file (*.msh) and then save the file.

• Click on Mesh on the left-side tree.
• In Details of Mesh, Quality, Mesh Metric and select the quality parameter to be evaluated. A histogram detailing the quality parameter and the number of elements inside the ranges is exhibited. Also, the summary of the Mesh Metric is exhibited in the left-side tree. Three of the main mesh quality parameters are detailed following:
  ○ Skewness: quantifies how close to ideal a face or a cell is. Values between 0 and 0.5 are recommended by Ansys [20].
• The mesh quality can be improved using the maximum Skewness as a target. In Quality, Target Skewness, set the value to 0,5, set Smoothing to High and click on Update.
• Check if the Skewness target was achieved (by clicking on the histogram bar, the elements are highlighted in the Graphics view – Fig. 5.a–b).
  • Element Quality: hybrid quality metric accounting for a relationship between the element area and the edge length. Values close to 1 are recommended.
  • Orthogonal Quality: quantifies the element orthogonality. Values close to 1 are recommended.

**Fig. 6:**
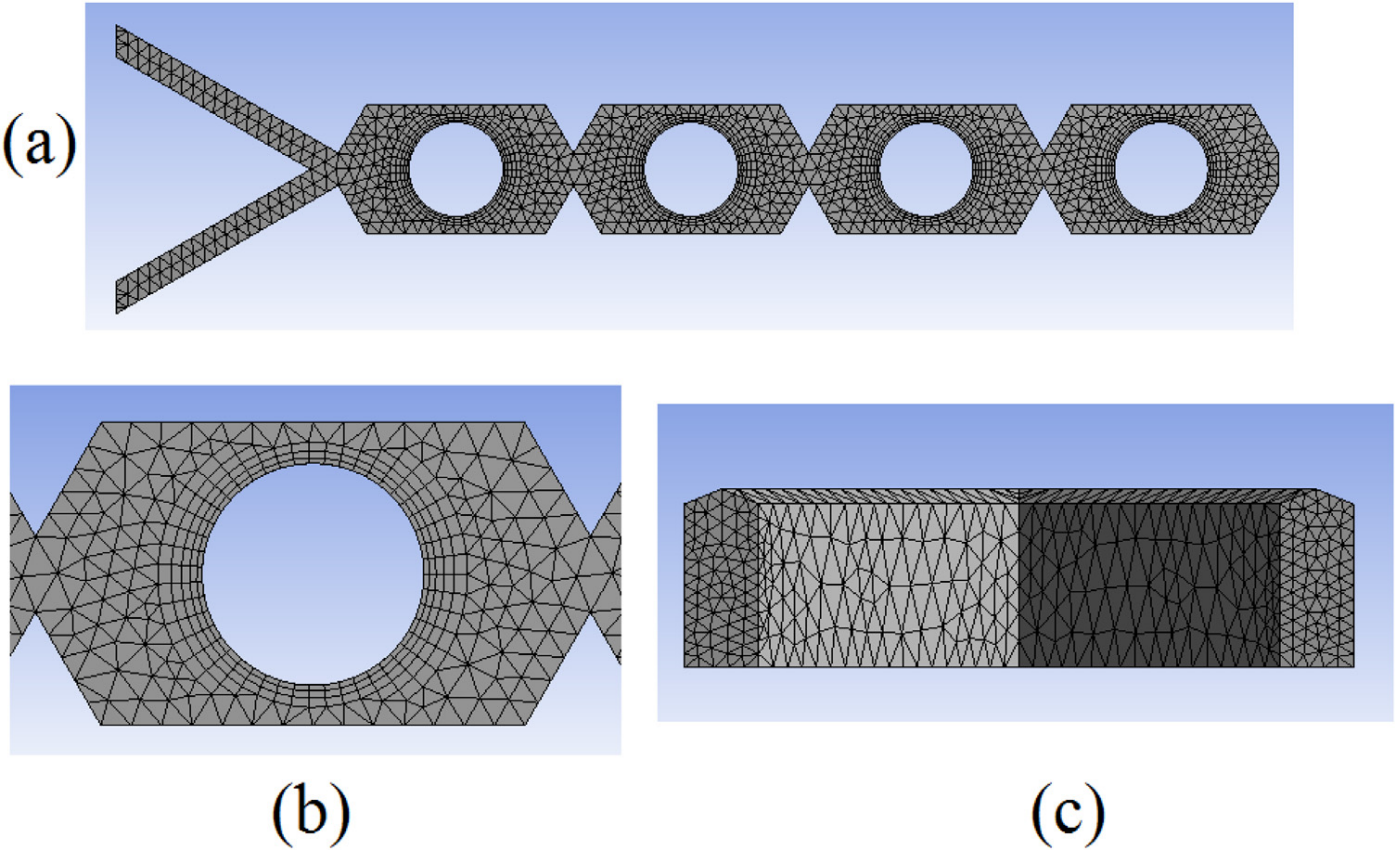
(a) Superior view of the entire mesh; (b) details of the prism layer zone generate around the circular obstacles; (c) details of the Y-inlet mesh.
• Proceed the meshing setup procedure in order to create at least two more refined meshes to perform the Grid Convergence Index (GCI) procedure [7].

Another geometry design was used to compare the devices performance. The MT microchannel has a triangular cross section (equilateral triangle shape) with 200 μm (edge size). The geometry was used to demonstrate the methodology application in different geometry and size scales. The MT file, in. step format, can be downloaded for free in Supplementary material 2.

### Pre-Processing

#### Fluid mixing setup

**Fig. 7:**
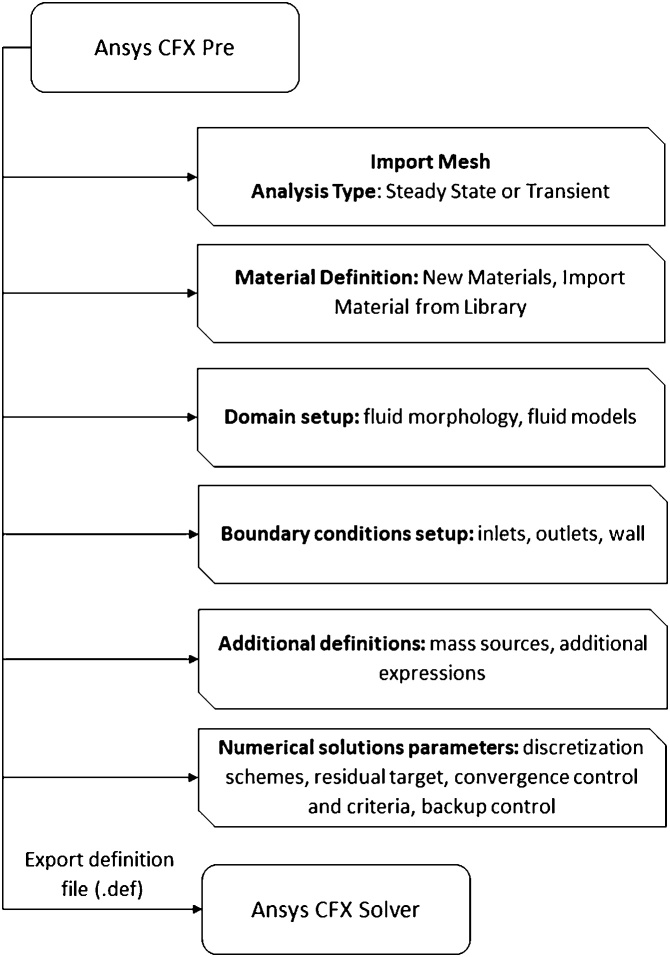
Flowchart of the second part of pre-processing: definition of physical modeling and numerical parameters.

The fluid mixing study was based on the mixture of ethanol and sunflower oil. The mathematical model consists in a nonreactive multicomponent single-phase, steady-state flow, assuming isothermal (25 °C), incompressible and laminar flow regime conditions. The setup procedure is described below. The flowchart presented in Fig. 7 summarizes the main steps of mathematical and numerical setup.

• Open Ansys CFX Pre.
• Import the mesh using the option FLUENT (.msh file type) and check out the size unit (for this example, in meter).
• In Analysis Type: Steady State.

##### Fluid and system definition

• Oil (creating a new material): right click on Materials, Insert Materials, Name: Sunflower Oil. In Basic Setting Tab, Option select Pure Substance, check the Thermodynamic State box and select Liquid. In material properties, insert thermodynamic properties (molar mass, 879.23 kg kmol^−1^, and density, 914.96 kg m^-3^) and transport properties (only dynamic viscosity, 0.0549 Pa s^−1^, for isothermal cases).
• Ethanol (importing an existing material from database): right click on Materials, Import Library Data, Liquid Phase Combustion and click on C2H6Ol and then OK (thermodynamic and transport properties are given at 25 °C).
• Creating a binary fluid mixture: right click on Materials, Insert Materials, Name: Mixture1. In Basic Setting Tab, Option select Variable Composition Mixture and check the Thermodynamic State box and select Liquid. In Material List select Sunflower Oil and C2H6Ol.
• In Default Domain, Basic Settings tab, Fluid and Particle Definitions, in Material, select the Mixture1 as Continuous Fluid. In Buoyancy Model select Buoyant Option and define the axis for gravity and the reference density, for example as the ethanol density (786 kg m^−3^). The reference pressure is defined by 1 atm.
• In Default Domain, Fluid Models tab, in Heat Transfer select Isothermal at 25 °C, in Turbulence select None (Laminar). In Component Models, select the option Transport Equation for C2H6Ol and check the Kinematic Diffusivity box, inserting its value. For the Sunflower oil select the option Constraint. The remaining settings are default

##### Boundary conditions

• Ethanol Feed Stream: Insert, Boundary, name as Inlet1. In Basic Setting tab, Boundary Type: Inlet, in Location select the corresponding boundary (inlet 1). In Boundary Details, Mass and Momentum, select the option Normal Speed, providing a prescribed value according to the Reynolds number. In Component Details, set the C2H6Ol mass fraction to 1 (pure ethanol stream).
• Oil Feed Stream: Insert, Boundary, name as Inlet2. In Basic Setting tab, Boundary Type: Inlet, in Location select the corresponding boundary (inlet 2). In Boundary Details, Mass and Momentum, select the option Normal Speed, providing a prescribed value according to the Reynolds number. In Component Details, set the C2H6Ol mass fraction to 0 (pure oil stream).
• Outlet: Insert, Boundary, name as Outlet. In Basic Setting tab, Boundary Type: Outlet, in Location select the corresponding boundary (outlet). In Boundary Details, Mass and Momentum, select the option Average Static Pressure, Relative Pressure = 0. The remaining settings are default
• Walls: Insert, Boundary, name as Wall. In Basic Setting tab, Boundary Type: Wall, in Location select the corresponding boundary (walls). In Boundary Details, Mass and Momentum, select the option No Slip Wall.

Table 2 summarizes the inlet velocities used in fluid mixing simulations.

**Table 2:** Inlet velocities used as boundary conditions in fluid mixing studies.

	MTB	MT
Reynolds Number	U_inlet_ (m/s)	
0.01	0.0003332	0.0052
0.1	0.003332	0.05196
1	0.033315	0.5196
10	0.33315	5.1962
50	1.6658	25.9808
100	3.3315	51.9615

##### Numerical details and output control

• In Solver Control, select the High Resolution advection scheme. In Convergence Control, set the Max. Iterations to 5000, Timescale Control to Auto Timescale, Aggressive with a Timescale Factor of 2.5. In Convergence Criteria set the Residual Type to RMS, with a target of 1 × 10^−5^ and a Conservation Target of 0.01. A target of RMS = 1 × 10^−5^ is recommended for good convergence and is satisfactory for most of engineering applications. A target of RMS = 1 × 10^-6^ or lower is recommended when simulating geometrically sensitive problems and in such cases double precision solver should be used. The conservation target is defined to ensure conservation in global balances. The default recommended value is 0.01, i.e., the global balance is obtained within 1 % of accuracy [20].
• In Output Control, in Backup tab, create a Backup Results, with Standard and Default option. In Output Frequency select an Iteration Interval of 50. The remaining settings are default.
• Save the. def (definition file), and run it in the Ansys CFX Solver.

##### Running the solver

• Open the CFX-Solver Manager 19 from the laucher.
• File > Define Run
• In Solver Input File, open the. def, previously saved.
• In Parallel Environment > Run mode > Intel MPI Local Parallel: Partitions = 4 (number of used cores can be defined in accordance with the computational resource available)
• In Working Directory the directory where the files will be written is defined.

**Fig. 8:**
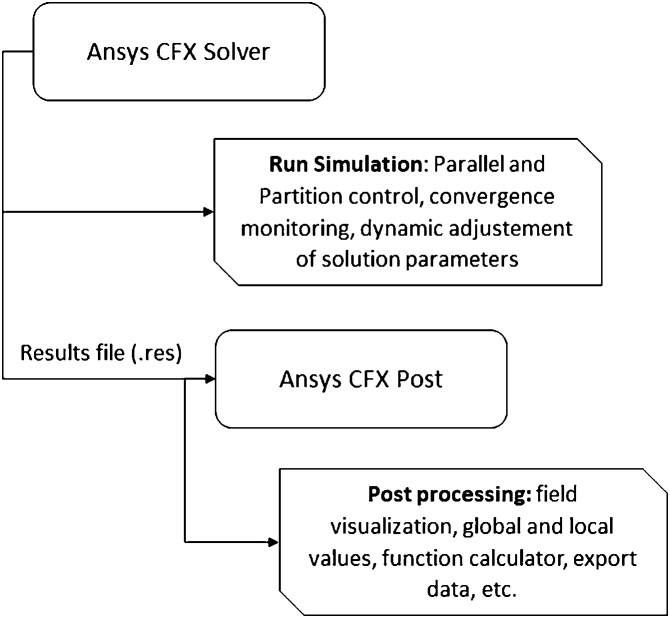
Flowchart of numerical solver and post-processing steps.

Fig. 8 depicts the flowchart of numerical solution and post-processing steps.

#### Chemical reaction setup

The chemical reaction study was based on sunflower oil transesterification with excess ethanol and sodium hydroxide catalyst, according to Santana et al. [14]. The mathematical model consists in a reactive multicomponent single-phase steady-state flow, assuming isothermal, incompressible and laminar flow regime conditions. The global reaction rate was considered. The operating conditions are 50 °C, ethanol/oil molar ratio of 9 and 1 % wt. NaOH. The setup procedure is described below.

• Open Ansys CFX Pre.
• Import the mesh using the option FLUENT (.msh file type) and check out the size unit.
• In Analysis Type: Steady State.

##### Fluid and system definition

• Creating the Vegetable Oil: right click on Materials, Insert Materials, Name: Sunflower Oil. In Basic Setting Tab, Option select Pure Substance, check the Thermodynamic State box and select Liquid. In material properties, insert thermodynamic properties (molar mass, 879.23 kg kmol^−1^, and density, 899.4 kg m^-3^) and transport properties (only dynamic viscosity, 0.0213 Pa s^−1^, for isothermal cases).
• Creating the Ethanol: right click on Materials, Import Library Data, Liquid Phase Combustion and click on C2H6Ol and then OK. Once thermodynamic and transport properties are given at 25 °C, In material properties, change thermodynamic property (density, 763 kg m^−3^) and transport property (dynamic viscosity, 0.000688 Pa s^-1^).
• Creating the Glycerol: right click on Materials, Insert Materials, Name: Glycerol. In Basic Setting Tab, Option select Pure Substance, check the Thermodynamic State box and select Liquid. In material properties, insert thermodynamic properties (molar mass, 92.08 kg kmol^−1^, and density, 1244.3 kg m^-3^) and transport properties (dynamic viscosity, 0.1465 Pa s^−1^).
• Creating the Biodiesel: right click on Materials, Insert Materials, Name: Glycerol. In Basic Setting Tab, Option select Pure Substance, check the Thermodynamic State box and select Liquid. In material properties, insert thermodynamic properties (molar mass, 308.45 kg kmol^−1^, and density, 861.01 kg m^-3^) and transport properties (dynamic viscosity, 0.003 Pa s^−1^).
• Creating the Inert (constraint): right click on Materials, Insert Materials, Name: Inert. In Basic Setting Tab, Option select Pure Substance and check the Thermodynamic State box and select Liquid. In material properties, insert thermodynamic properties (molar mass, 879.23 kg kmol^−1^, and density, 899.4 kg m^-3^) and transport properties (dynamic viscosity, 0.0213 Pa s^−1^).
• Creating a multicomponent fluid mixture: right click on Materials, Insert Materials, Name: Mixture. In Basic Setting Tab, Option select Variable Composition Mixture and check the Thermodynamic State box and select Liquid. In Material List select Sunflower Oil, C2H6Ol, Glycerol, Biodiesel and Inert.
• In Default Domain, Basic Settings tab, Fluid and Particle Definitions, in Material, select the Mixture as Continuous Fluid. In Buoyancy Model select Buoyant Option and define the axis for gravity and the reference density, for example as the inert/oil density (899.4 kg m^−3^). The reference pressure is defined by 1 atm.
• In Default Domain, Fluid Models tab, in Heat Transfer select Isothermal at 50 °C, in Turbulence select None (Laminar). In Component Models, select the option Transport Equation for Biodiesel, C2H6Ol, Glycerol and Oil. Check the Kinematic Diffusivity boxes for these species, inserting their values. For the Inert select the option Constraint. The remaining settings are default.

##### Boundary conditions

• Ethanol Feed Stream: Insert, Boundary, name as Inlet1. In Basic Setting tab, Boundary Type: Inlet, in Location select the corresponding boundary (inlet 1). In Boundary Details, Mass and Momentum, select the option Normal Speed, providing a prescribed value according to the residence time. In Component Details, set the Sunflower Oil mass fraction to 1 (pure oil stream). Set the mass fraction of further components to zero.
• Oil Feed Stream: Insert, Boundary, name as Inlet2. In Basic Setting tab, Boundary Type: Inlet, in Location select the corresponding boundary (inlet 2). In Boundary Details, Mass and Momentum, select the option Normal Speed, providing a prescribed value according to the residence time. In Component Details, set the Ethanol mass fraction to 1 (pure oil stream). Set the mass fraction of further components to zero.
• Outlet: Insert, Boundary, name as Outlet. In Basic Setting tab, Boundary Type: Outlet, in Location select the corresponding boundary (outlet). In Boundary Details, Mass and Momentum, select the option Average Static Pressure, Relative Pressure = 0. The remaining settings are default
• Walls: Insert, Boundary, name as Wall. In Basic Setting tab, Boundary Type: Wall, in Location select the corresponding boundary (walls). In Boundary Details, Mass and Momentum, select the option No Slip Wall.

Table 3 summarizes the inlet velocities used in chemical reaction simulations.

**Table 3:** Inlet velocities used as boundary conditions in chemical reaction analysis.

Residence time (s)	MTB		MT	
	Oil	Ethanol	Oil	Ethanol
	U_inlet_ (cm/s)		U_inlet_ (cm/s)	
5	1.3132	0.7300	1.2892	0.7167
10	0.6566	0.3650	0.6446	0.3583
30	0.2189	0.1217	0.2149	0.1194
60	0.1094	0.0608	0.1074	0.0597
120	0.0547	0.0304	0.0537	0.0299

##### Mass sources due to the chemical reaction

• Insert Subdomain, in Basic Setting Tab select the fluid domain (mesh volume) in Location.
• In Sources tab, check the Sources box.
• In Equation Sources, check the Biodiesel.mf box, Option: Source, in Source click on Enter Expression, Sbio.
• In Equation Sources, check the C2H6Ol.mf box, Option: Source, in Source click on Enter Expression, Sethanol.
• In Equation Sources, check the Glycerol.mf box, Option: Source, in Source click on Enter Expression, Sglycerol.
• In Equation Sources, check the Oil.mf box, Option: Source, in Source click on Enter Expression, Soil.
• Right-click on Expressions, Insert, Expression, Name Sbio, click OK.
• In Details of Sbio, Definition tab insert the following expression: Biodiesel.Molar Mass*(3*rTG)
• Right-click on Expressions, Insert, Expression, Name Sethanol, click OK.
• In Details of Sethanol, Definition tab insert the following expression: C2H6Ol.Molar Mass*(-3*rTG)
• Right-click on Expressions, Insert, Expression, Name Sglycerol, click OK.
• In Details of Sglycerol, Definition tab insert the following expression: Glycerol.Molar Mass*(rTG)
• Right-click on Expressions, Insert, Expression, Name Soil, click OK.
• In Details of Soil, Definition tab insert the following expression: Sunflower Oil.Molar Mass*(-rTG)
• Right-click on Expressions, Insert, Expression, Name rTG, click OK.
• In Details of rTG, Definition tab insert the following expression: kdir*Oleo.Molar Concentration*C2H6Ol.Molar Concentration-kinv*Biodiesel.Molar Concentration*Glicerol.Molar Concentration
• Right-click on Expressions, Insert, Expression, Name kdir, click OK.
• In Details of kdir, Definition tab insert the following expression: 15.68e-6 [m^3 mol^-1 s^-1]
• Right-click on Expressions, Insert, Expression, Name kinv, click OK.
• In Details of kinv, Definition tab insert the following expression: 9.42e-8 [m^3 mol^-1 s^-1]

##### Numerical details and output control

• Use the similar definitions described in 2.1.3.
• Save the. def (definition file), and run it in the Ansys CFX Solver.
• Proceed to the solver running (Section 2.1.4)

* *For more details of post-processing, it is recommended for the reader to consult the tutorials provided by ANSYS, CFD-Post User's Guide [available online at:* https://bit.ly/2Xakmx7] or videos on YouTube, for example, the available at: https://bit.ly/2Llhh66

### Methodology application and results

#### Independence mesh test by GCI methodology

In order to demonstrate the reported methodology application in two geometries (MT and MTB designs) were developed for fluid mixing and chemical reaction flow systems. MT design presents triangular cross section, an uncommon shape for microdevices, with equilateral triangle side of 200 μm and 35 mm of longitudinal length. MTB geometry was based on Santana et al. [14] with scale-up to millimeter size (width =5000 μm; height =3000 μm; longitudinal length =35 mm). Table 4 summarizes the GCI index results based on mixing (*M*) and performance (*PI*) indexes. The subscripts *1*, *2* and *3* represent refined, intermediate and coarse meshes, the subscripts 21 and 32 denote for the refinement ratios fine/intermediate and intermediate/coarse meshes, respectively, *N* is the number of elements, *r* is the refinement factor, *Φ* is the evaluated property (simulation prediction), *p* is the apparent order, *ext* stands for the extrapolated value, *e_a_* is the approximate relative error, *e_ext_* is the extrapolate relative error and *GCI_fine_* is the discretization uncertainty of the fine grid or the fine grid convergence index.

**Table 4:** GCI analysis based on Mixing and Performance Indexes.

	MTB	MT
*N_1_; N_2_; N_3_*	259,118; 189,096; 118,675	621,489; 343,351; 224,424
*r_21_*	1.3703	1.8101
*r_32_*	1.5934	1.5299
*Φ =* Mixing Index, *M*		
*Φ_1_*	0.9610	0.9908
*Φ_2_*	0.9640	0.9900
*Φ_3_*	0.9503	0.9979
*p*	3.4504	5.3983
*Φ_ext_^21^*	0.9594	0.9908
*e_a_^21^*(%)	0.32	0.08
*e_ext_^21^*(%)	0.16	0.00
***GCI_fine_21*(%)**	**0.20**	**0.00**
*Φ =* Performance Index, *PI*		
*Φ_1_*	4.4752 × 10^−6^	1.7413 × 10^−8^
*Φ_2_*	4.7321 × 10^−6^	1.8828 × 10^−8^
*Φ_3_*	4.6760 × 10^−6^	1.9565 × 10^−8^
*p*	3.4944	1.9879
*Φ_ext_^21^*	4.3471 × 10^−6^	1.6785 × 10^−8^
*e_a_^21^* (%)	5.74	8.12
*e_ext_^21^*(%)	2.95	3.74
***GCI_fine_21*(%)**	**3.58**	**4.51**

For MTB geometry, mesh refinements of 259,118, 189,096, 118,675 elements were generated. The GCI indexes for fine mesh were 0.2 % and 3.58 %, respectively for mixing and performances indexes. The higher value for *PI* can be attributed to the pressure drop. However, the spatial discretization uncertainty was relative low (below 4 %). For MT geometry numerical grids ranging from 224,424 to 654,492 elements were generated. For this geometry, a very low GCI index for *M* was observed, increasing for *PI*. Since *M* is based in a statistical analysis, the mesh refinement increases the number of samples used to estimate *M*, minimizing the errors associated with spatial discretization. In contrast, *PI* also depends on the pressure drop along the channel, resulting in larger discretization uncertainty. The GCI index approaching zero in MT was related to the extrapolated value of *M* was equal to the predicted by the fine mesh. According to Table 4, the fine meshes were able to capture the fluid flow behavior with good accuracy with spatial discretization associated errors below 5 %.

#### Microdevices performance for fluid mixing and chemical reaction

**Fig. 9:**
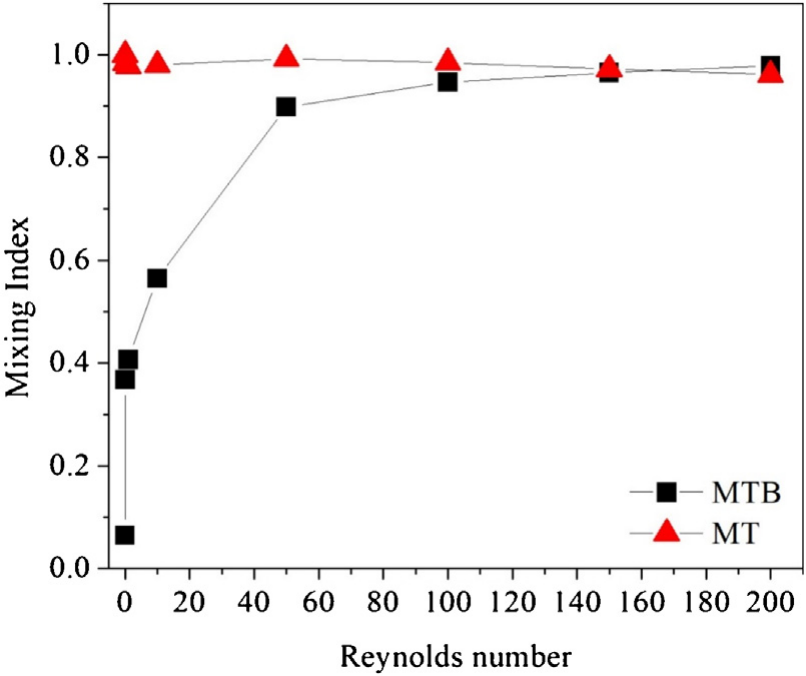
Mixing index as a function of Reynolds number for the proposed geometries: MTB design: micromixer with triangular baffles and circular obstructions; MT design: micromixer with triangular cross section.

The mixing and performance indexes evaluation was performed for a Reynolds number range from 0.01 to 200. MT geometry exhibited mixing index near to 1 for all range of Reynolds number evaluated, while MTB presented a superior mixing performance (*M* ≈ 0.98) at Re = 200, and the lowest mixing index at Re = 0.01 (*M* = 0.06) (Fig. 9). MT geometry provided mixing index virtually constant for the range of Reynolds number evaluated, while the mixing index increased with Reynolds number in MTB design.

**Fig. 10:**
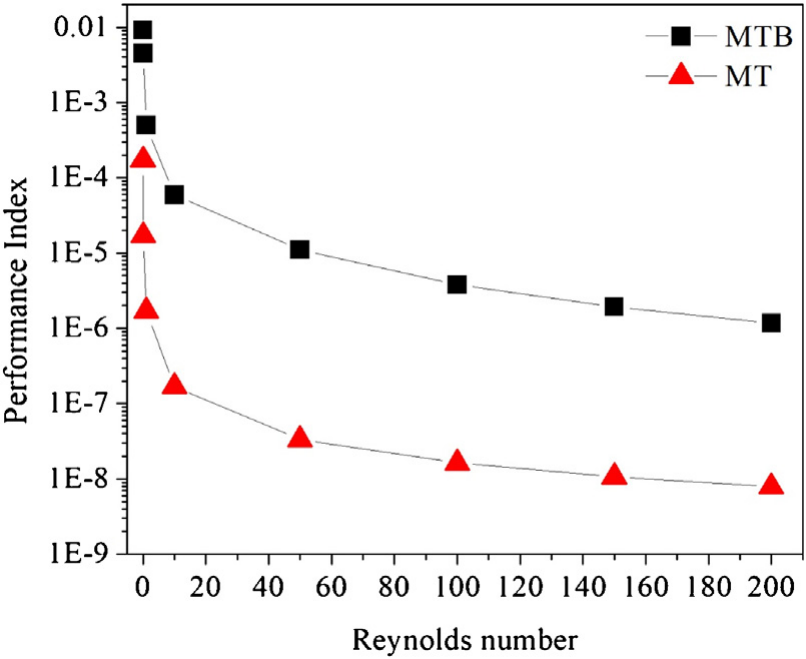
Performance index as a function of Reynolds number for the proposed geometries: MTB design: micromixer with triangular baffles and circular obstructions; MT design: micromixer with triangular cross section.

The performance index (PI) is given by the ratio of mixing index to the unit pressure drop. High values of PI represent good micromixer performance. Fig. 10 presents the PI obtained from the simulations. MTB exhibited superior performance at Re = 0.01 (PI ≈ 9.2 × 10^−3^) and at Re = 200, the PI decrease to 1.18 × 10^-6^. For MT design, the PI for Re = 0.01 and 200 were 1.72 × 10^-4^ and 7.86 × 10^-9^, respectively. The PI decreased with increment of Reynolds number, since pressure drop is directly proportional to flow rate in laminar flow regime.

**Fig. 11:**
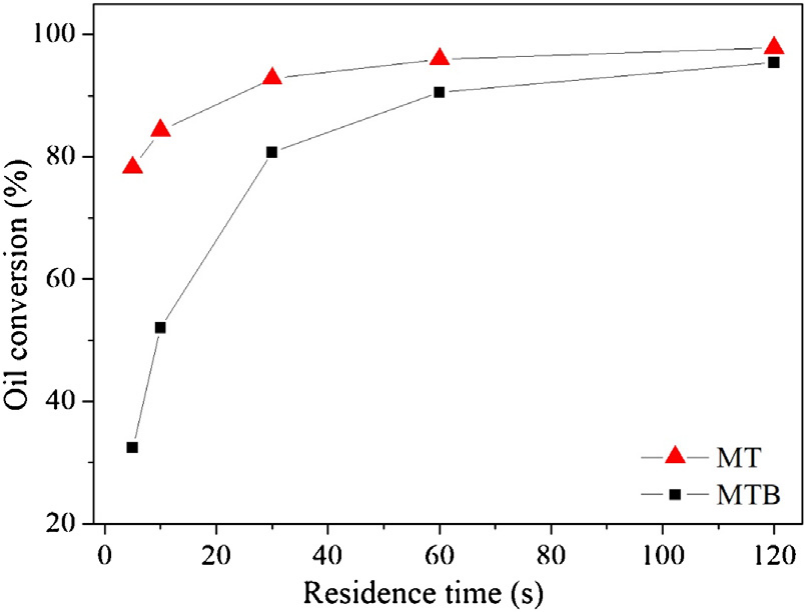
Oil conversion as a function of Residence time for the proposed geometries: MTB design: micromixer with triangular baffles and circular obstructions; MT design: micromixer with triangular cross section.

The proposed methodology was also applied in a chemical reaction flow system, specifically in biodiesel synthesis from vegetable oil. Fig. 11 presents the effect of fluids residence time in oil conversion for the two proposed designs. Both devices exhibited high maximum oil conversions, 95.42 % (MTB) and 97.82 % (MT). The size scale affected the reactor performance mostly in short residence times (32.43 % (MTB) and 78.18 % (MT), for a residence time of τ = 5 s).

### Discussion and conclusions

Numerical simulations allow fast development of microdevices resulting in reduction of time and costs in project. CFD can also provide great detailing of flow field in severe operating conditions. The microdevice developer can evaluate different designs and assess its performance previously to the physical prototype manufacturing for experimental runs. The reported methodology can be employed in a wide range of chemical reaction and fluid mixing flow systems.

The fluid mixing and biodiesel synthesis from sunflower oil/ethanol was evaluated in two geometries: MT, micromixer with triangular cross section (in micrometer scale) and MTB, micromixer with triangular baffles and circular obstructions (in millimeter scale). Both micromixer designs exhibited high mixing indexes (M ≥ 0.98). The fluid flow in microscale tends to occur in laminar regime, due to the low Reynolds numbers. In this condition, the chemical species mixing takes place by molecular diffusion due to the absence of fluid macroscale mixing, however with smaller molecular paths, i.e., the mean free path to interact with another molecule, result in more effective molecular interaction, enhancing the mixing performance. Since MT has microscale dimensions (triangle lateral =200 μm), a molecule in the channel center experiences a 100 μm diffusion path to interact with distant molecules (in wall region), in contrast, in MTB this molecular path was 2500 μm.

**Fig. 12:**
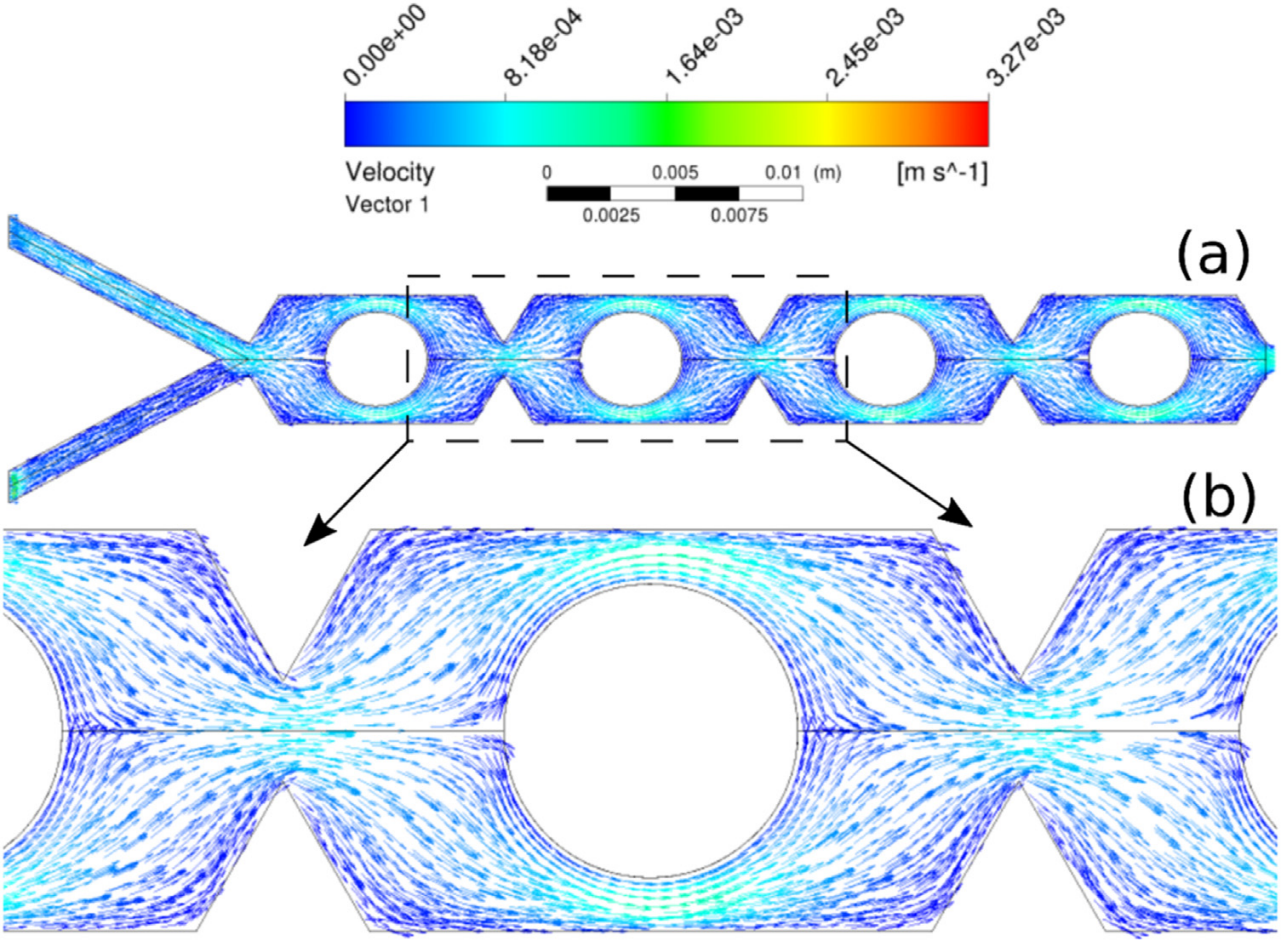
Velocity vector field for Re = 0.01: (a) along MTB device; (b) details of obstacles upstream and downstream flow pattern.

**Fig. 13:**
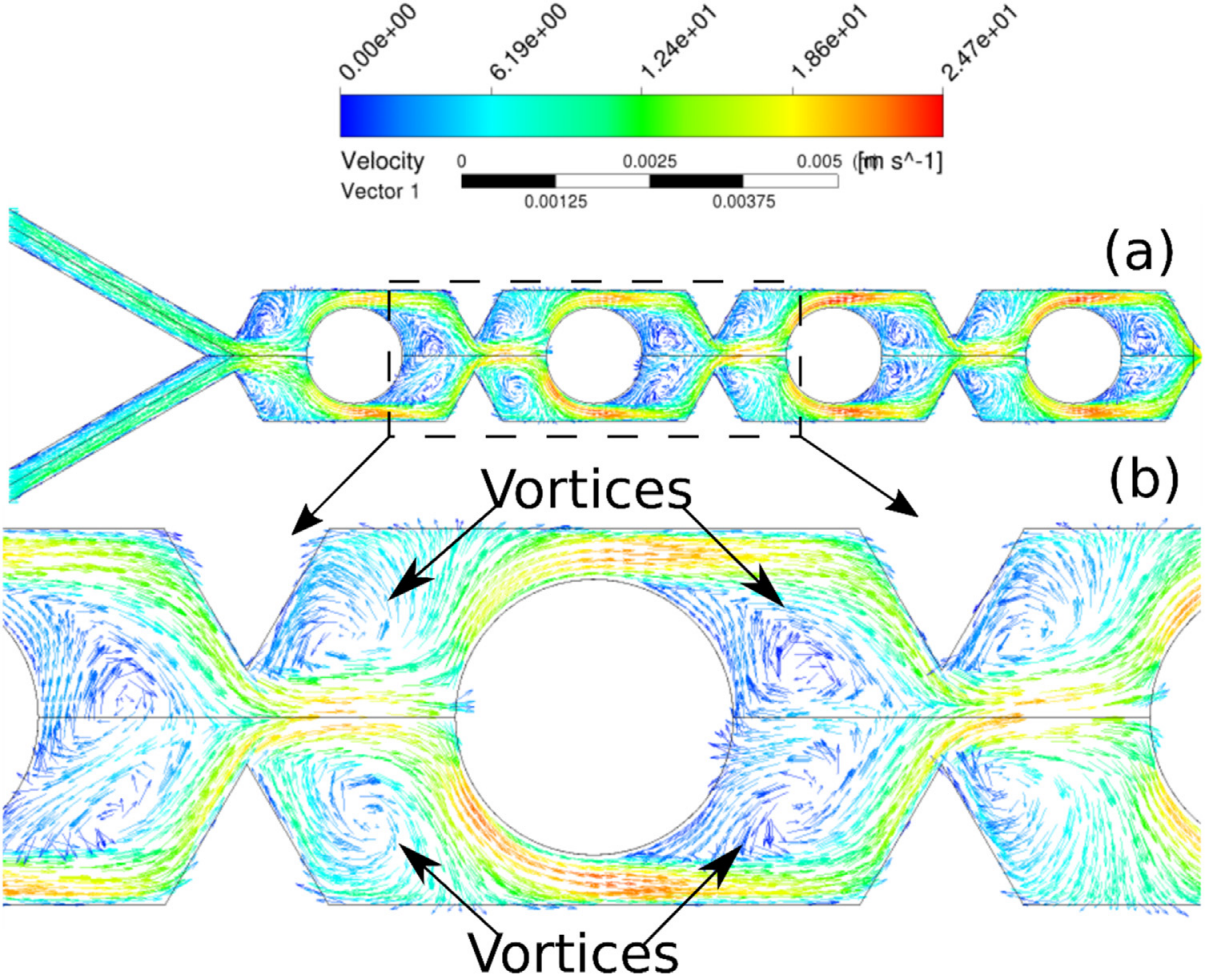
Velocity vector field for Re = 200: (a) along MTB device; (b) details of vortex generation in obstacles upstream and downstream zones.

The flow rate increment, and consequently, the Reynolds number, introduces other mixing mechanisms, including chaotic advection, related to the phenomena in which a simple Eulerian velocity field could lead to a chaotic response for the distribution of a Lagrangean marker [23], i.e., the dynamic system driving the trajectory is chaotic and consequently, resulting trajectory is strongly sensible to initial conditions. Chaotic advection promotes flow disturbance increasing the interactions among chemical species and then enhancing the mixing process. Chaotic advection also induces secondary transport increasing local rate of mass transfer. In addition, the circular obstacles inside MTB channel enhanced the mass transfer, as reported by Santana et al. [14]. The circular obstacles change the flow direction, inducing vortex formation. In MTB design the vortex generation process depends on the Reynolds number, i.e., the flow velocity (Fig. 12). At Re = 0.1, the fluid smoothly bypassed the obstacle, i.e., no fluid detachment from obstacle wall was observed (Fig. 12.a,b), and fluid mixing occurred mostly by channel throttling (convergence zone) and division of main flow in substreams [14]. From Re = 50 was observed vortex zones, i.e., regions of adverse flow, increasing local mass transfer. As reported by Santana et al. [14], vortex generation was observed in obstacles upstream and downstream regions (Fig. 13.a,b).

The mixing behavior is directly related to the predominant mechanism of mass transfer. Due to the micrometric scale in MT design, the diffusion path is reduced, resulting in enhanced mixing even for very low Reynolds numbers. For low Reynolds numbers (usually, below 10), the molecular diffusion prevails. The increment of Reynolds number increased the convective transport influence on fluid mixing (as clarified by the flow patterns shown in Fig. 12, Fig. 13).

As previously stated, the increment of Reynolds number decreased the PI in both geometries, due to the increase of pressure drop with the substantial velocity increment. MTB design exhibited superior performance regarding MT. This result is attributed to the reduced pressure drop in milliscale compared to microscale. MTB pressure drop ranged from 7.03 Pa (Re = 0.01) to 8.33 × 10^5^ Pa (Re = 200), while, MT experienced pressure drops of 17.85 Pa (Re = 0.01) to 1.22 × 10^8^ Pa (Re = 200). In addition, considering the mixing index from two designs (M ≈ 0.98 in both geometries at Re = 200), implies that MTB design provided a superior global performance.

The sunflower oil transesterification with ethanolic solution of sodium hydroxide was evaluated, once the oil conversions is directly related to the mixing degree between the fluids [24], allowing the performance assessment of micro- and milli-reactors. In the previous section the increase on the oil conversion with the residence time up to a maximum value for both designs was noticed. In longer residence times, molecules of triglyceride and ethanol experience enough time to interact and react, thus the chemical reaction will be virtually complete at channel outlet [14]. Furthermore, longer residence times favor molecular diffusion, resulting in greater uniformity of chemical species along the channel. This behavior was confirmed from Fig. 11 for residence time ranging from 10 s to 120 s.

Finally, the reported methodology allowed the evaluation of fluid mixing (based on the quantification of mixing index) and fluid mixing accompanied by homogeneous chemical reaction. The methodology can be used by experienced or beginners users of CFD packages. We expect that the reported methodology contributes to the popularization of CFD usage among researchers, scientist and Microfluidics enthusiasts, and that future studies aims to optimize the microdevices by numerical simulations, becoming the device development procedure faster and less expensive.
